# Erratum

**Published:** 2010-12

The November Science Selection article “Disinfection By-products and Bladder Cancer: Common Genetic Variants May Confer Increased Risk” [ Environ Health Perspect 118:A491 (2010)] incorrectly stated that “Among individuals who carried both of the *GSTT1* and *GSTZ1* genotypes noted above (28% of study participants), those with the highest DBP exposure were at a 1.5 times increased risk of bladder cancer compared with carriers with the lowest DBP exposure.” Among individuals who carried both of the *GSTT1* and *GSTZ1* genotypes noted above (28% of study participants), those with the highest DBP exposure actually were at a 5.9 times increased risk of bladder cancer compared with carriers with the lowest DBP exposure. Risk was only 1.5 times higher with high versus low DBP exposure among study participants who lacked both genotypes. *EHP* regrets the error.

